# Association of admission aggregate index of systemic inflammation with lower-extremity deep vein thrombosis after severe trauma

**DOI:** 10.1371/journal.pone.0360039

**Published:** 2026-10-21

**Authors:** Qichang Sun, Donghui Bian

**Affiliations:** Trauma Center, The 960th Hospital of the Joint Logistics Support Force of the Chinese People’s Liberation Army, Jinan, Shandong, China; University of Health Sciences, Beyhekim Training and Research Hospital, TÜRKIYE

## Abstract

Early identification of patients at risk for lower-extremity deep vein thrombosis (LEDVT) after severe trauma remains difficult. This retrospective cohort study evaluated whether admission aggregate index of systemic inflammation (AISI), derived from platelet, neutrophil, monocyte, and lymphocyte counts, was associated with ultrasound-confirmed in-hospital LEDVT detected before the earlier of discharge or post-injury day 30 and whether it improved risk stratification. Among 223 adults with an Injury Severity Score of 16 or higher and evaluable bilateral lower-extremity ultrasonography, 93 (41.7%) had LEDVT. Each 1-SD increase in log-transformed AISI was associated with LEDVT after adjustment for age, sex, Injury Severity Score, Glasgow Coma Scale score, and shock index (odds ratio [OR], 2.07; 95% CI, 1.47–2.91; P < 0.001) and after additional adjustment for D-dimer, fibrin/fibrinogen degradation products, prothrombin time, activated partial thromboplastin time, and fibrinogen (OR, 1.86; 95% CI, 1.26–2.73; P = 0.002). Adding AISI to clinical variables increased the area under the receiver operating characteristic curve (AUC) from 0.619 to 0.714 (AUC difference, 0.095; bootstrap 95% CI, 0.023 to 0.168; P = 0.009). After coagulation markers were included, the AUC difference was smaller and uncertain (0.018; 95% CI, −0.010 to 0.046; P = 0.166), although the Brier score improved by 0.014 (P = 0.020). For Model 4, bootstrap-corrected and repeated-cross-validation AUCs were 0.797 and 0.790, and calibration slopes were 0.831 and 0.773, respectively. With 93 events and 11 candidate parameters, these internally validated results indicate shrinkage and require prospective external validation; AISI should be interpreted as a complementary admission marker.

## Introduction

Lower-extremity deep vein thrombosis (LEDVT) is frequently detected after severe trauma, but reported rates and risk factors for post-traumatic venous thromboembolism (VTE) vary across major-trauma cohorts [1–4]. Early decisions about lower-extremity venous ultrasonography and thromboprophylaxis are complicated by evolving bleeding risk, injury pattern, operative needs, and interruptions or delays in pharmacologic prophylaxis [5–7]. These factors make early risk estimation clinically relevant, particularly for selecting patients for closer ultrasound surveillance and repeated reassessment of prophylaxis eligibility.

Despite this need, early thrombotic risk stratification after severe trauma remains difficult. Clinical variables such as injury severity, Glasgow Coma Scale score, and shock index describe injury burden and physiologic derangement, but they do not directly characterize early cellular inflammatory or platelet-related changes. Conventional coagulation markers, including D-dimer, fibrin/fibrinogen degradation products, prothrombin time, activated partial thromboplastin time, and fibrinogen, are routinely available, but trauma itself alters coagulation and fibrinolysis through tissue injury, hemorrhage, surgery, and resuscitation [8,9]. In this setting, fibrin-turnover markers may be broadly elevated and correlated with other coagulation parameters, limiting their ability to separate patients with and without LEDVT early after admission.

Venous thrombosis involves innate immune cells, platelets, and the endothelium in addition to coagulation activation. Experimental models have shown that monocytes, neutrophils, and platelets cooperate during venous thrombus initiation and propagation, and that neutrophil extracellular traps can promote deep vein thrombosis [10,11]. Reviews have also described neutrophil extracellular traps, platelet-leukocyte interactions, and sterile inflammatory signaling as components of immunothrombosis [12–14]. This framework is relevant to severe trauma, in which inflammation, endothelial injury, platelet activation, and venous stasis may coexist, although these mechanisms are not directly measured in most retrospective clinical cohorts.

The aggregate index of systemic inflammation (AISI) is a derived complete-blood-count ratio that combines platelet, neutrophil, monocyte, and lymphocyte counts. AISI was initially operationalized as one of several inflammatory cell indices in an elective thoracic-surgery cohort [15]; it was not derived or prospectively validated as a trauma-specific diagnostic score. Subsequent studies evaluated AISI and related cellular indices in severe trauma, hospitalized trauma or surgical cohorts, and orthopedic trauma [16–19]. Those reports support biological and prognostic interest, but they do not constitute prospective validation of AISI for trauma LEDVT screening. We therefore examined a narrower incremental question: whether admission AISI added information after clinical severity measures and routinely measured coagulation markers were considered, including when initial-ultrasound-positive and isolated intramuscular thromboses were handled separately.

Accordingly, we evaluated the association between admission AISI and ultrasound-confirmed in-hospital LEDVT detected before the earlier of discharge or post-injury day 30 after severe trauma. We also examined whether AISI added risk-stratification information beyond early clinical variables and conventional coagulation parameters. Secondary analyses compared AISI with related immune-inflammatory indices and individual blood-cell components, and sensitivity analyses excluded initial-ultrasound-positive LEDVT and isolated intramuscular venous thrombosis.

## Materials and methods

### Study design and setting

This single-center retrospective cohort study was conducted at the 960th Hospital, a tertiary referral trauma center. Consecutive adult patients with severe trauma admitted between January 2021 and January 2026 were screened. Severe trauma was operationally defined as an Injury Severity Score (ISS) of 16 or higher. Reporting followed the Strengthening the Reporting of Observational Studies in Epidemiology (STROBE) statement [20]. S1 Checklist is the completed 22-item cohort-study reporting checklist and identifies where each item is addressed. Patient selection is summarized in Fig 1.

**Fig 1:**
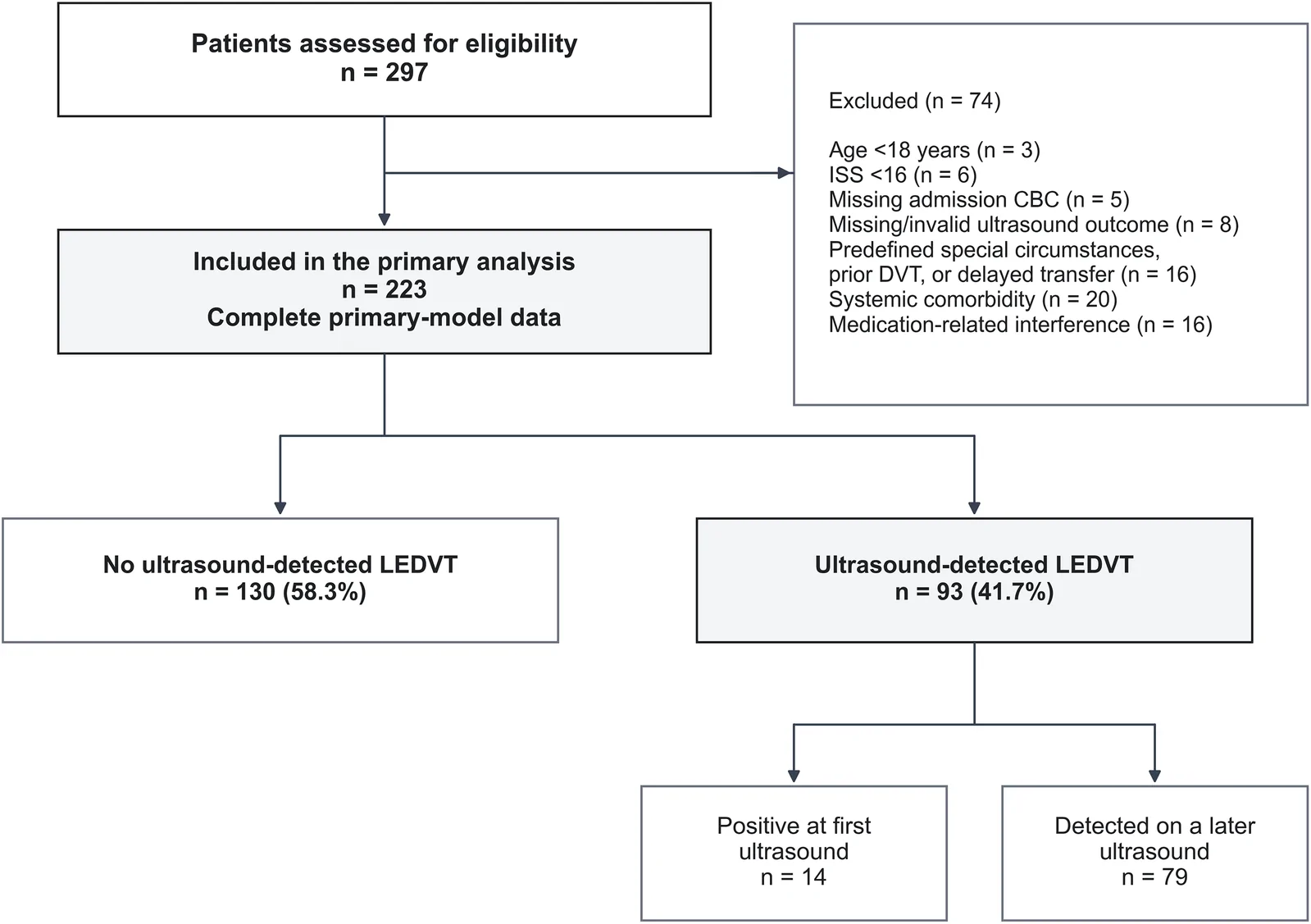
Study flow diagram. Of 297 screened patients, 223 met all eligibility criteria and had evaluable bilateral lower-extremity ultrasonography.

Ethics statement: The study was approved by the Ethics Committee of the 960th Hospital (approval No. 2026−178), with a waiver of informed consent because of its retrospective design. All data were obtained from the electronic medical record system and de-identified before analysis. Patient data were accessed for research purposes on 6 May 2026. Authorized clinical investigators had access to information that could identify individual participants during data extraction; all data were de-identified before analysis, and the analysis dataset used for statistical analysis contained no direct identifiers.

### Data integrity and quality control

The final analytical dataset comprised 223 eligible patients. All outcome and predictor fields used in the primary models were extracted from the electronic health record. During quality control, examination-count fields for 23 records were re-abstracted from the original ultrasound reports; archived entries were retained in audit columns. A sensitivity analysis excluded these 23 records.

### Study population

Patients were eligible if they were 18 years or older, met the operational severe-trauma definition (ISS ≥ 16), underwent bilateral lower-extremity venous ultrasonography during hospitalization, and had complete blood-count and coagulation results within 24 hours after admission.

Patients were excluded for prior DVT; invalid bilateral lower-extremity ultrasound outcome data; delayed transfer precluding baseline assessment; pregnancy; predefined special clinical conditions; systemic comorbidity likely to affect inflammation or thrombosis; immunosuppressive therapy; pre-injury therapeutic anticoagulation; age below 18 years; ISS below 16; or missing admission laboratory data. The four categories comprising the 22 unmet eligibility or insufficient-outcome-data exclusions were age below 18 years, ISS below 16, missing admission complete blood count, and missing valid bilateral lower-extremity ultrasound outcome.

Patients with LEDVT detected on the first post-admission ultrasound examination were included in the primary analysis because the study concerned early inpatient risk stratification rather than strictly incident thrombosis after a documented negative baseline scan. A prespecified sensitivity analysis excluded these 14 patients.

### Outcome definition and ultrasound assessment

The primary outcome was ultrasound-confirmed lower-extremity deep vein thrombosis (LEDVT) detected before the earlier of hospital discharge or post-injury day 30. No post-discharge event was classified as a primary outcome. Bilateral lower-extremity venous ultrasonography was targeted early after admission and repeated for symptoms, high-risk status, delayed or interrupted pharmacologic prophylaxis, or clinician concern; it was not performed on a fixed research schedule. Examination day was treated as detection time, not biological onset time. The study did not systematically screen upper-extremity veins and did not capture upper-extremity or catheter-associated DVT.

For the primary analysis, LEDVT included proximal, distal, isolated intramuscular, and mixed lower-extremity deep venous thrombosis. Initial-ultrasound-positive cases were included in the primary analysis and excluded in a sensitivity analysis. A second sensitivity analysis excluded isolated intramuscular venous thrombosis.

### Data collection and immune-inflammatory indices

Clinical, laboratory, ultrasound, and treatment data were obtained from the hospital electronic health record. Baseline variables included age, sex, ISS, Glasgow Coma Scale (GCS) score, admission vital signs, and shock index. Thromboprophylaxis formed part of routine inpatient care, with modality and timing individualized according to bleeding risk, injuries, procedures, and contraindications. Mechanical ventilation, surgery, and pharmacologic prophylaxis were recorded during hospitalization and were evaluated only in sensitivity analyses because they were post-baseline processes. Mechanical prophylaxis comprised bilateral intermittent pneumatic compression or sequential compression devices (IPC/SCD), elastic stockings or an intermittent pneumatic pump, or contralateral IPC/SCD when the injured limb was locally contraindicated. Pharmacologic prophylaxis used low-molecular-weight heparin (predominantly enoxaparin) or unfractionated heparin and was initiated or resumed after reassessment of hemostasis, bleeding risk, operative needs, and contraindications, consistent with references 5–7 and 23–27. Interruption and resumption intervals were directly abstracted from medication-administration records and operative timelines and were evaluated only in sensitivity analyses.

### Additional covariates and longitudinal data

Additional patient-level variables comprised pelvic or lower-extremity fracture, early activity, bedbound days, prophylaxis timing, total hospital length of stay, and serial ultrasound assessments. These variables were directly abstracted from original structured electronic-record fields, imaging reports, treatment records, or screening logs. Range and chronology discrepancies were resolved by re-review of the original source documents, with archived values retained for auditability. All examinations recorded for the 223-patient primary cohort occurred during hospitalization; no post-discharge event was included in the primary outcome.

Laboratory variables were defined using the first available results within 24 hours after admission. Complete blood count variables included neutrophil count, lymphocyte count, monocyte count, and platelet count. Conventional coagulation variables included prothrombin time, activated partial thromboplastin time, fibrinogen, D-dimer, and fibrin/fibrinogen degradation products (FDP).

The systemic immune-inflammation index (SII), systemic inflammation response index (SIRI), and aggregate index of systemic inflammation (AISI) were calculated from the first complete blood count obtained within 24 hours:


$$\begin{array}{c} SII=platelet\;count\times neutrophil\;count/lymphocyte\;count \end{array}$$

$$\begin{array}{c} SIRI=neutrophil\;count\times monocyte\;count/lymphocyte\;count \end{array}$$

$$\begin{array}{c} AISI=platelet\;count\times neutrophil\;count\times monocyte\;count/lymphocyte\;count \end{array}$$

AISI was the prespecified primary cellular index; SII and SIRI were exploratory comparators.

### Statistical analysis

Continuous variables are presented as mean (SD) or median [IQR], as appropriate, and categorical variables as counts and percentages. To avoid treating descriptive screening comparisons as multiple confirmatory tests, Table 1 reports no baseline P values. Instead, it reports LEDVT-minus-no-LEDVT absolute differences with 2,000-resample bootstrap 95% CIs and standardized mean differences (SMDs). These descriptive measures were not used to select covariates for the prespecified models.

**Table 1:** Selected characteristics according to LEDVT status.

Characteristic	Overall	No LEDVT	LEDVT	Difference (95% CI)	SMD
Age, years	56.22 (16.49)	54.95 (17.32)	58.00 (15.17)	3.05 (−1.17 to 6.94)	0.19
Male sex	165/223 (74.0%)	95/130 (73.1%)	70/93 (75.3%)	2.2 (−10.3 to 13.9) pp	0.05
Shock index	0.79 (0.21)	0.77 (0.21)	0.83 (0.20)	0.06 (0.00 to 0.11)	0.27
Injury Severity Score	24.44 (6.65)	23.68 (6.36)	25.52 (6.93)	1.84 (0.05 to 3.57)	0.28
Glasgow Coma Scale	11.46 (3.50)	12.03 (3.16)	10.67 (3.80)	−1.36 (−2.30 to −0.44)	0.40
Mechanical ventilation	89/223 (39.9%)	42/130 (32.3%)	47/93 (50.5%)	18.2 (5.8 to 31.3) pp	0.38
ICU length of stay, days	17.67 [12.84, 22.36]	15.98 [11.40, 20.18]	20.48 [15.18, 25.32]	4.50 (2.03 to 6.58)	0.62
Neutrophils, 10^9/L	10.54 (4.43)	9.85 (3.28)	11.52 (5.54)	1.67 (0.45 to 2.90)	0.38
Lymphocytes, 10^9/L	0.63 [0.46, 0.89]	0.63 [0.46, 0.89]	0.64 [0.44, 0.90]	0.01 (−0.10 to 0.13)	0.06
Monocytes, 10^9/L	0.75 [0.57, 1.00]	0.69 [0.53, 0.93]	0.85 [0.65, 1.12]	0.16 (0.07 to 0.29)	0.54
Platelets, 10^9/L	164.31 (69.92)	146.64 (46.01)	189.01 (88.16)	42.37 (23.38 to 63.07)	0.63
AISI	1764.02 [897.84, 3141.34]	1389.99 [883.19, 2169.55]	2431.68 [1031.57, 4421.64]	1041.69 (392.47 to 1659.69)	0.44
Fibrinogen, g/L	3.35 (1.51)	3.10 (1.27)	3.70 (1.74)	0.60 (0.17 to 1.02)	0.40
D-dimer, µg/mL	8.60 [3.72, 19.88]	8.55 [3.07, 20.53]	8.60 [4.10, 18.77]	0.05 (−3.49 to 4.09)	0.08
FDP, mg/L	30.12 [13.15, 73.85]	31.63 [12.62, 82.43]	30.10 [13.74, 62.09]	−1.53 (−15.66 to 12.11)	0.16

*Values are mean (SD), median [IQR], or n/N (%). Difference is LEDVT minus no LEDVT; continuous-variable CIs were obtained by 2,000 bootstrap resamples. SMD, standardized mean difference; pp, percentage points.*

Because SII, SIRI, AISI, D-dimer, and FDP were right-skewed, they were natural log-transformed and then standardized before regression modeling. Odds ratios are reported per 1-SD increase in the transformed variable.

The analysis was designed to assess the incremental risk-stratification value of AISI rather than to derive a final clinical prediction score. Multivariable logistic regression was used to evaluate the association between AISI and LEDVT. Four prespecified models were fitted. Model 1 included age, sex, ISS, GCS score, and shock index. Model 2 added AISI to Model 1. Model 3 added conventional coagulation parameters to Model 1, including D-dimer, FDP, prothrombin time, activated partial thromboplastin time, and fibrinogen. Model 4 added AISI to Model 3. The main incremental comparison was Model 4 versus Model 3; Model 2 versus Model 1 was considered a secondary comparison.

The study size was fixed by the number of consecutive eligible patients identified during the prespecified January 2021 to January 2026 inclusion period; no a priori sample-size calculation was used. Model 4 contained 11 candidate parameters excluding the intercept for 93 outcome events, corresponding to 8.45 events per candidate parameter. No stepwise or outcome-guided variable selection was performed, and model complexity was considered when interpreting internal validation.

Model discrimination was assessed using the area under the receiver operating characteristic curve (AUC), calibration using plots, intercept, and slope, and overall prediction error using the Brier score. Internal validation used 2,000 bootstrap resamples and repeated stratified 5-fold cross-validation (20 repeats; 100 test folds). All model fitting, log transformation, standardization, and prediction steps were repeated within each bootstrap sample or the model-development subset of each cross-validation split. Bootstrap resampling also estimated 95% CIs and two-sided P values for paired changes in AUC and Brier score. Decision-curve analysis and integrated discrimination improvement were exploratory.

Sensitivity analyses excluded patients with initial-ultrasound-positive LEDVT, excluded isolated intramuscular venous thrombosis, additionally adjusted for in-hospital mechanical ventilation, surgery, and pharmacologic prophylaxis, restricted analysis by surveillance strategy, added primary injury site, and excluded all 23 records whose ultrasound examination counts were re-abstracted from the original reports. Additional models adjusted for pelvic or lower-extremity fracture, early activity, bedbound days, prophylaxis timing, total hospital length of stay, and ultrasound timing. A restricted cubic spline was used as an exploratory AISI functional-form diagnostic. All secondary, sensitivity, functional-form, decision-curve, and supplementary analyses were exploratory; no multiplicity-adjusted confirmatory claim was made.

### Missing data and software

The final 223-patient cohort had complete values for the outcome and all variables in the four prespecified models; therefore, complete-case analysis removed no additional included patient and multiple imputation was not performed. The screening report did not retain patient-level fields sufficient to compare every exclusion with the included cohort. A limited age, sex, and ISS comparison was performed for patients excluded because of missing data, but this comparison does not establish that the missingness mechanism was random.

Analyses were performed in Python 3.10.4 using pandas 2.3.3, NumPy 2.2.6, SciPy 1.15.3, statsmodels 0.14.6, and scikit-learn 1.7.2. The random seed was 20260726. All model tests were two-sided; P < 0.05 was used for the prespecified primary association and incremental comparisons. Secondary, sensitivity, and supplementary analyses were interpreted as exploratory, without multiplicity adjustment; descriptive Table 1 comparisons were not assigned P values.

### Use of artificial intelligence tools

OpenAI Codex was used during revision to assist with English-language editing, document formatting, figure preparation, spreadsheet presentation, and cross-file consistency checks. The authors independently verified all medical-record abstractions, statistical results, references, tables, figures, and supporting files. The tool was not used to generate, impute, or alter clinical data or as an independent source of scientific evidence. The authors reviewed and approved all changes and take full responsibility for the submitted work.

## Results

Of 297 screened patients, 74 were excluded: 3 were younger than 18 years, 6 had an ISS below 16, 5 lacked an admission complete blood count, 8 lacked a valid bilateral lower-extremity ultrasound outcome, 16 had special circumstances, prior DVT, or delayed transfer, 20 had systemic comorbidities likely to affect inflammation or thrombosis risk, and 16 had medication-related interference. The final cohort therefore comprised 223 patients (Fig 1).

Within the inpatient assessment window ending at the earlier of discharge or day 30, 93 patients (41.7%) had ultrasound-confirmed LEDVT and 130 (58.3%) did not. The first ultrasound was performed at a median of day 3 [IQR, 2–5]. Among LEDVT cases, the recorded detection day was 9 [6–14]; 14 (15.1%) were detected on the initial examination and 36 (38.7%) were isolated intramuscular thromboses. The median last negative or positive examination day was 13 [7–19]. After re-abstraction of 23 examination-count fields from the original ultrasound reports, the median number of examinations was 2 [2,3]. Outcome and surveillance distributions are shown in Fig 2.

**Fig 2:**
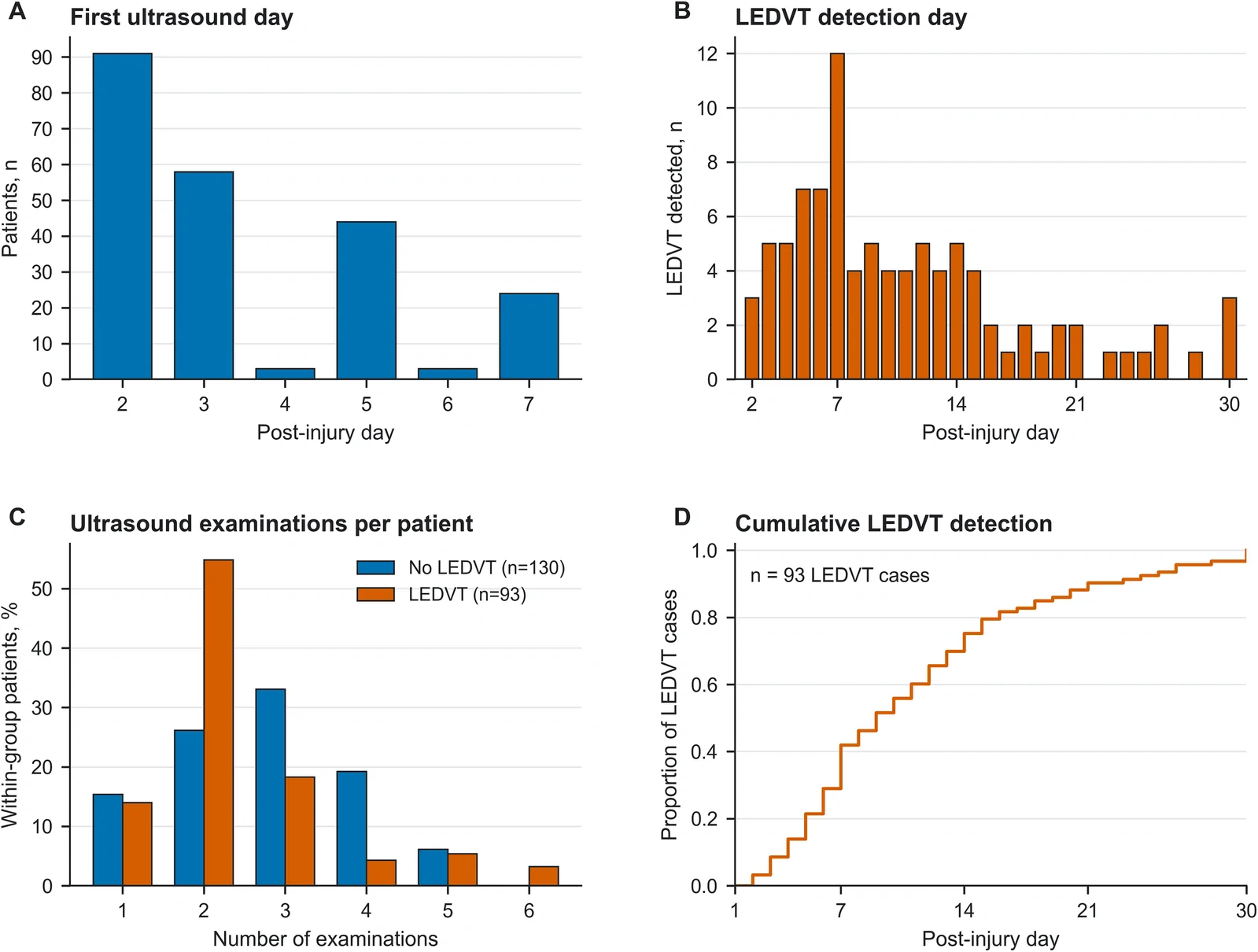
Ultrasound timing and surveillance. (A) Distribution of the first ultrasound day among all 223 patients. (B) Distribution of the LEDVT detection day among the 93 affected patients. (C) Number of ultrasound examinations per patient, shown as within-group percentages for the 130 no-LEDVT and 93 LEDVT groups. (D) Cumulative proportion of the 93 LEDVT cases detected by post-injury day. Examination and detection days are observation times; the cumulative curve is not a biological incidence curve.

Selected baseline characteristics are shown in Table 1. The largest absolute standardized differences were observed for platelet count (SMD 0.63), ICU length of stay (0.62), monocyte count (0.54), AISI (0.44), neutrophil count (0.38), fibrinogen (0.40), GCS score (0.40), and mechanical ventilation (0.38). Admission D-dimer and FDP showed small standardized differences. In the observed source fields, 209/223 patients (93.7%) received mechanical prophylaxis: bilateral IPC/SCD in 158, elastic stockings or an intermittent pneumatic pump in 25, and contralateral IPC/SCD when the injured limb was locally contraindicated in 26; 14 (6.3%) had no mechanical prophylaxis recorded. Pharmacologic prophylaxis was recorded for 176/223 patients (78.9%): standard LMWH/enoxaparin in 139, dose-adjusted LMWH in 9, and UFH in 28. Its first recorded start day was post-injury day 2 [IQR, 1–4; range, 0–6]. These values are descriptive and were not used for data-driven covariate selection.

In multivariable logistic regression, each 1-SD increase in log-transformed AISI was associated with higher odds of LEDVT after adjustment for age, sex, ISS, GCS score, and shock index (OR, 2.07; 95% CI, 1.47–2.91; P < 0.001). After additional adjustment for D-dimer, FDP, prothrombin time, activated partial thromboplastin time, and fibrinogen, the association remained (OR, 1.86; 95% CI, 1.26–2.73; P = 0.002) (Table 2).

**Table 2:** Association between admission AISI and LEDVT in primary and observed-data sensitivity analyses.

Analysis	N	Events,n	AISI OR (95% CI)	P value	AUC	Brier
Clinical + AISI	223	93	2.07 (1.47–2.91)	<0.001	0.714	0.208
Clinical + coagulation + AISI	223	93	1.86 (1.26–2.73)	0.002	0.829	0.158
Exclude initial-ultrasound-positive LEDVT	209	79	1.83 (1.22–2.74)	0.003	0.823	0.154
Exclude isolated intramuscular LEDVT	187	57	2.30 (1.40–3.79)	0.001	0.862	0.125
Add observed ventilation, surgery, prophylaxis	223	93	1.91 (1.28–2.86)	0.002	0.852	0.149
Exclude 23 records with re-abstracted examination counts	200	70	1.87 (1.17–2.97)	0.008	0.851	0.139

Odds ratios are per 1-SD increase in ln(AISI). The 23-record sensitivity excludes all records whose ultrasound examination-count fields were re-abstracted from the original reports; corrections to examination counts did not change outcomes or other clinical variables.

Model performance is summarized in Table 3. Adding AISI to the clinical model increased the AUC from 0.619 to 0.714 (AUC difference, 0.095; bootstrap 95% CI, 0.023 to 0.168; P = 0.009) and reduced the Brier score by 0.023 (95% CI, 0.005–0.040; P = 0.015). Adding AISI to the clinical-plus-coagulation model increased the AUC from 0.811 to 0.829, but the AUC difference was smaller and uncertain (0.018; 95% CI, −0.010 to 0.046; P = 0.166). The Brier score improved by 0.014 (95% CI, 0.002 to 0.025; P = 0.020), and the likelihood-ratio test was P < 0.001. For Model 4, the bootstrap-corrected AUC/Brier score were 0.797/0.178, with calibration intercept −0.062 and slope 0.831; repeated-cross-validation values were 0.790/0.179, with intercept −0.085 and slope 0.773. With 93 events and 11 candidate parameters excluding the intercept (8.45 events per candidate parameter), slopes below 1 were interpreted as evidence of optimism and a need for shrinkage and external validation.

**Table 3:** Performance and internal validation of the prespecified models.

Model	Apparent AUC (95% CI)	Apparent Brier	Bootstrap correctedAUC / Brier / intercept / slope	Repeated CVAUC / Brier / intercept / slope	Incremental comparison
M1: clinical	0.619 (0.540–0.690)	0.231	0.577 / 0.244 / −0.083 / 0.731	0.558 / 0.243 / −0.172 / 0.489	
M2: clinical + AISI	0.714 (0.641–0.783)	0.208	0.683 / 0.222 / −0.050 / 0.839	0.671 / 0.221 / −0.081 / 0.748	AUC difference 0.095 (0.023 to 0.168); P = 0.009
M3: clinical + coagulation	0.811 (0.751–0.864)	0.172	0.778 / 0.191 / −0.061 / 0.835	0.768 / 0.191 / −0.085 / 0.786	
M4: clinical + coagulation + AISI	0.829 (0.770–0.882)	0.158	0.797 / 0.178 / −0.062 / 0.831	0.790 / 0.179 / −0.085 / 0.773	AUC difference 0.018 (−0.010 to 0.046); P = 0.166

*For each validation method, values are AUC / Brier / calibration intercept / calibration slope. Repeated CV used repeated stratified 5-fold cross-validation with 20 repeats (100 test folds); bootstrap optimism correction used 2,000 resamples. Incremental P values are paired bootstrap estimates. Calibration slopes below 1 indicate overfitting/shrinkage and are not evidence of external validity.*

Apparent receiver operating characteristic curves and calibration plots are shown in Fig 3.

**Fig 3:**
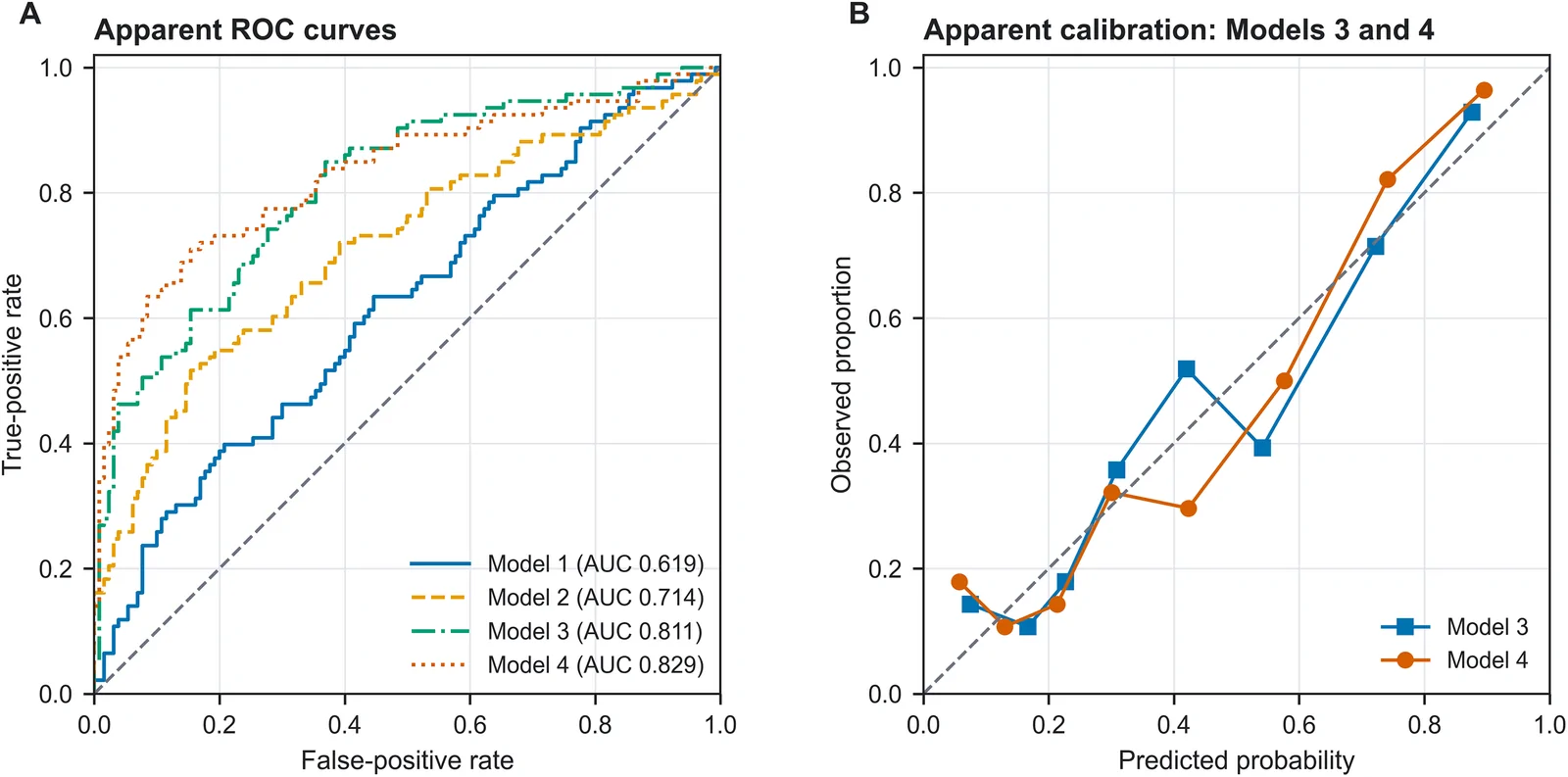
Incremental discrimination and apparent calibration. (A) Receiver operating characteristic curves for Models 1–4. (B) Apparent calibration curves for Models 3 and 4; points show grouped observed risks and lines show model-based calibration. Apparent performance is shown here; optimism-corrected and repeated-cross-validation performance is reported in the text, Table 3, and S2 Appendix.

Exploratory sensitivity analyses are shown in Table 2 and Fig 4. AISI remained associated with LEDVT after excluding initial-ultrasound-positive cases (OR, 1.83; 95% CI, 1.22–2.74), excluding isolated intramuscular thrombosis (OR, 2.30; 95% CI, 1.40–3.79), adding ventilation, surgery, and pharmacologic prophylaxis (OR, 1.91; 95% CI, 1.28–2.86), and excluding all 23 records whose examination counts were re-abstracted from the original ultrasound reports (OR, 1.87; 95% CI, 1.17–2.97). Estimates were similar after adding pelvic or lower-extremity fracture (OR, 1.84), fracture plus bedbound status and ventilation (OR, 1.94), time-ordered prophylaxis (OR, 1.83), or ultrasound timing and examination count (OR, 1.81). These analyses were exploratory and were not adjusted for multiple comparisons.

**Fig 4:**
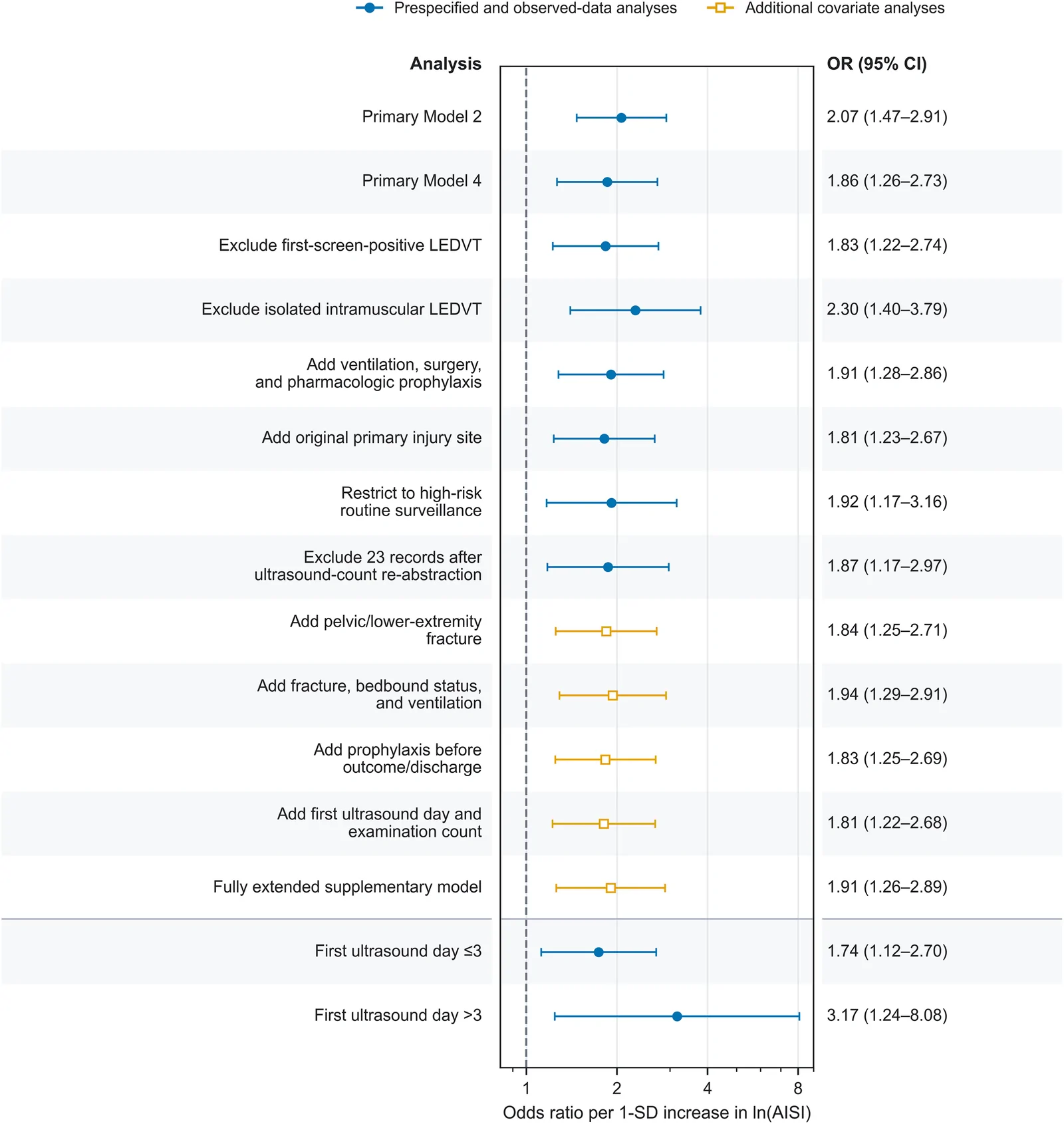
Sensitivity analyses for the association between admission AISI and LEDVT. Odds ratios are per 1-SD increase in log-transformed AISI. The forest plot separates prespecified and observed-data analyses from additional covariate analyses. Horizontal bars show 95% confidence intervals; model definitions and estimates are provided in S2 Appendix.

An exploratory 3-degree-of-freedom restricted cubic spline fit the data better than the prespecified linear ln(AISI) term (likelihood-ratio χ^2^ = 11.25; 2 df; P = 0.004; Fig 5). Because this non-multiplicity-adjusted functional-form analysis was exploratory and unstable at distributional extremes, the prespecified linear OR remains the main estimate; interpretation should focus on a graded, potentially nonlinear association rather than a constant effect or a clinical cutoff.

**Fig 5:**
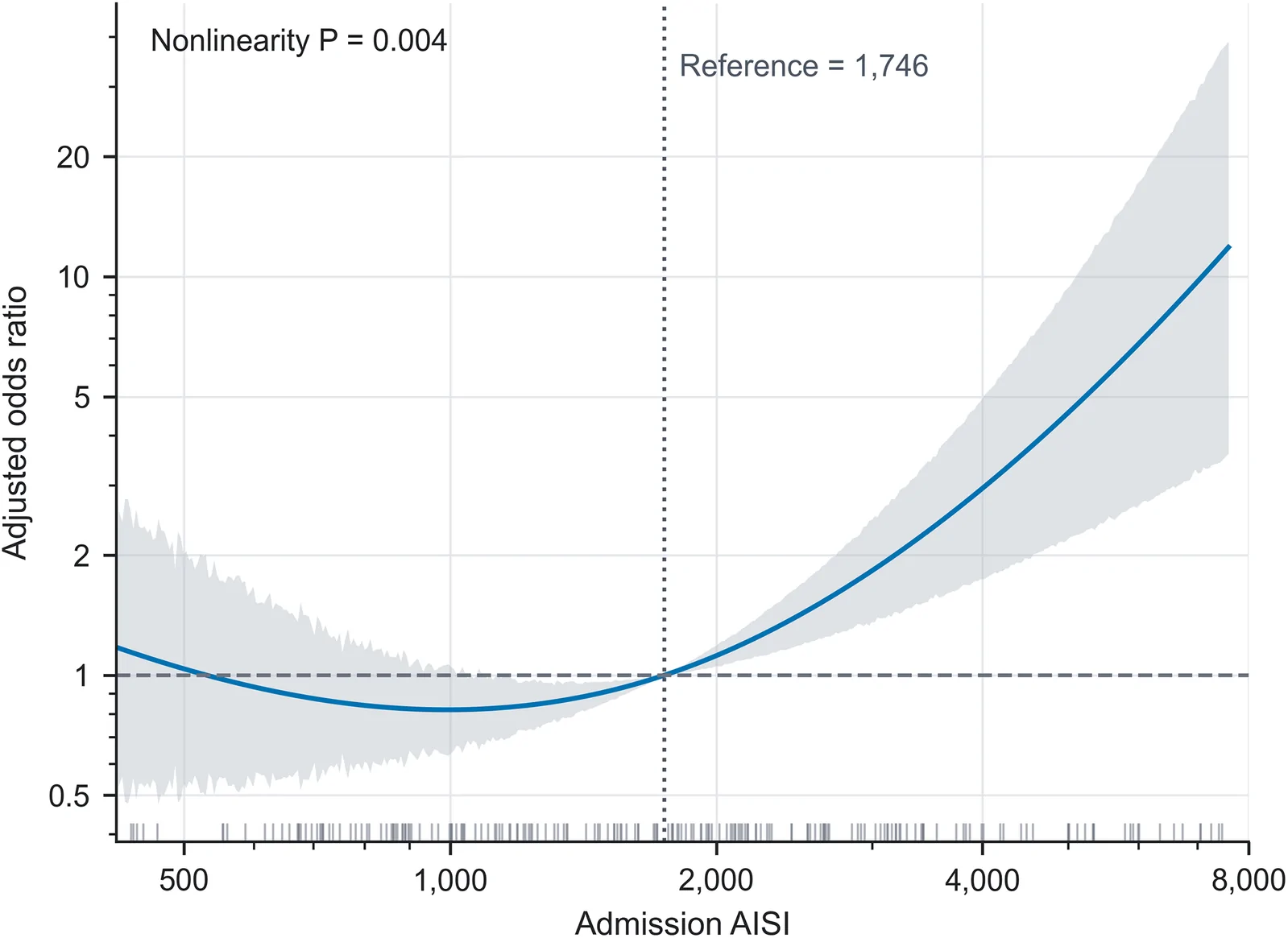
Restricted cubic spline analysis of admission AISI and LEDVT. The adjusted odds-ratio curve is referenced to AISI = 1,746 (the sample geometric mean). Shading shows the 95% confidence interval, and rug marks show the observed AISI distribution. The test for nonlinearity yielded P = 0.004; the spline does not define a validated clinical threshold.

Full model coefficients, candidate-parameter count and events-per-parameter summary, internal-validation AUC and Brier scores, calibration intercepts and slopes, decision-curve analysis, comparator-index analyses, coagulation diagnostics, ultrasound observation-process summaries, and all supplementary models are provided in S1 Data. Screening, missingness, length-of-stay and discharge, additional covariate, and 23-record re-abstraction quality-control summaries are compiled in S2 Appendix.

## Discussion

In this cohort of adults with severe trauma who underwent lower-extremity venous ultrasonography, admission AISI was associated with ultrasound-detected LEDVT before the earlier of discharge or post-injury day 30. The association persisted across prespecified observed-data sensitivity analyses, including exclusion of initial-ultrasound-positive cases and isolated intramuscular thrombosis. The results support continued evaluation of AISI as a complementary admission marker; they do not establish incident thrombosis, causality, or a treatment threshold.

The nested models clarified the limited incremental role of AISI. Adding AISI to clinical variables alone improved discrimination and prediction error. After conventional coagulation markers were included, the AUC increase was small and statistically uncertain, although the Brier score and model likelihood improved. For Model 4, bootstrap and repeated cross-validation reduced the apparent AUC of 0.829 to 0.797 and 0.790, and calibration slopes were 0.831 and 0.773. Together with 93 events for 11 candidate parameters, these results indicate residual optimism and the likely need for shrinkage; they do not constitute external validation. These findings argue against presenting AISI as a stand-alone predictor or as a replacement for clinical assessment and established prophylaxis decisions.

These findings are consistent with trauma studies in which post-injury VTE risk varies with injury pattern, physiologic derangement, immobilization, transfusion, prophylaxis, and surveillance strategy. Prior trauma studies have reported higher VTE rates in selected major-trauma populations and have identified injury burden, fracture pattern, neurologic impairment, transfusion, and surveillance strategy as associated factors [3,4,21,22]. Prevention remains challenging because timing, dosing, anti-Xa monitoring strategies, and system-level implementation differ across trauma settings [5–7,23–27]. In that context, this study addressed a narrower question: whether an admission cellular inflammatory index provides information beyond clinical and coagulation variables. The high LEDVT rate in this cohort should be interpreted in the context of the study design: patients had severe trauma, underwent routine or clinically indicated ultrasound surveillance, and the outcome included proximal, distal, intramuscular, and mixed thrombotic phenotypes. This rate is therefore not directly comparable with studies limited to symptomatic VTE, pulmonary embolism, or proximal DVT. Atabey and Kocaoglu reported very high apparent discrimination for AISI in 216 patients hospitalized for trauma or surgery with suspected DVT or pulmonary embolism (AUC 0.995 for DVT) [17]. The lower apparent AUC and more modest incremental gains in our consecutive severe-trauma cohort may reflect differences in case selection, outcome scope, ultrasound surveillance, and multivariable adjustment; direct performance comparison is therefore limited.

The laboratory pattern helps place the AISI association in context. Patients with LEDVT had higher AISI, SII, SIRI, neutrophil counts, monocyte counts, platelet counts, and fibrinogen levels, whereas admission D-dimer and fibrin/fibrinogen degradation products did not differ materially between groups. AISI combines several cell lines involved in inflammatory and platelet-related responses, and prior experimental and review work is compatible with a role for monocytes, neutrophils, platelets, extracellular traps, and the endothelium in venous thrombus formation [10–14]. A clinical trauma study linking postinjury platelet aggregation with VTE further supports the relevance of platelet-related signals in this setting [22]. After severe trauma, these processes may coexist with tissue injury, venous stasis, and acute-phase coagulation changes. The present data do not establish that AISI directly mediates thrombosis. AISI may instead mark patients with a stronger early inflammatory response, greater platelet availability, or more severe physiologic stress not fully reflected by ISS or routine coagulation markers.

The coagulation results should also be interpreted carefully. In the early post-trauma period, D-dimer and fibrin/fibrinogen degradation products may be elevated for reasons other than LEDVT, including tissue injury, hemorrhage, surgery, resuscitation, and fibrinolysis [8,9]. Their nonsignificant between-group differences and unstable adjusted estimates should not be interpreted as evidence that these markers are unrelated to post-traumatic thrombosis. Rather, these findings are compatible with collinearity, broad activation of coagulation and fibrinolysis after trauma, and limited specificity of fibrin-turnover markers in severely injured patients.

Any clinical interpretation of AISI should remain narrow. The present results do not support using AISI alone to start, withhold, or intensify anticoagulation. If replicated, it might be evaluated as one admission signal for closer reassessment and standardized ultrasound surveillance after bleeding risk is acceptable. A prospective, multicenter study should prespecify ultrasound time points, adjudicate incident lower- and upper-extremity VTE, capture fracture pattern, mobility, ventilation duration, transfusion, prophylaxis timing and interruptions, and follow patients after discharge.

The component analyses require caution. In exploratory analyses, platelet-only and all-four-component models showed higher apparent discrimination than the AISI model, and all components came from the same first admission complete blood count. However, these models allowed more flexible parameterization and were evaluated in a modest single-center cohort. Although cross-validation and bootstrap correction reduced the apparent performance, the platelet signal was not eliminated. These findings should therefore be viewed as exploratory. External cohorts are needed to determine whether platelet count alone, platelet-monocyte combinations, or AISI provides the most reproducible admission signal.

Several design features should be noted. The study used an ISS-based severe-trauma definition, admission laboratory measurements, prespecified nested models without stepwise or outcome-guided variable selection, transformation of skewed biomarkers, paired incremental-performance estimates, and bootstrap and repeated-cross-validation procedures. The study size was fixed by the number of consecutive eligible patients during the prespecified inclusion period; no a priori sample-size calculation was used. Sensitivity analyses addressed initial-ultrasound-positive LEDVT, isolated intramuscular thrombosis, major observed in-hospital treatments, surveillance strategy, primary injury site, and the 23 records with re-abstracted ultrasound counts.

The study has important limitations. First, it was retrospective and single-center, so causal inference and generalizability are limited. Residual confounding from detailed fracture anatomy, transfusion burden, immobilization, ventilation duration, prophylaxis timing or interruption, and clinician decision-making cannot be excluded. The treatment-adjusted and additional covariate-adjusted models were directionally consistent, but post-baseline adjustment is vulnerable to confounding by indication and the supplementary models remain exploratory.

Second, ultrasound timing and repetition reflected routine practice, symptoms, risk status, and clinician judgment rather than a fixed research protocol. A negative examination could therefore be followed by a later positive result because of interval thrombus formation or an earlier false-negative examination, and recorded detection day cannot identify biological onset. Median total hospital length of stay was 29 days, 109 of 223 patients remained hospitalized through day 30, and all documented examinations and events in the primary cohort were inpatient. Post-discharge ultrasound was not systematically available. The 114 patients discharged before day 30 were not assumed to remain event-free through day 30; discharge truncated observation and may have led to under-ascertainment of later LEDVT relative to patients who remained hospitalized. Consequently, the primary window ended at the earlier of discharge or post-injury day 30, and no post-discharge event was classified in the primary outcome.

Third, the outcome combined proximal, distal, isolated intramuscular, and mixed lower-extremity thromboses and did not include systematic upper-extremity or catheter-related DVT surveillance. Fourth, the exploratory spline suggested nonlinearity. Model 4 included 11 candidate parameters for 93 events (8.45 events per candidate parameter), and bootstrap and repeated-cross-validation calibration slopes of 0.831 and 0.773 indicate overfitting/shrinkage. Finally, internal validation cannot establish transportability; external prospective validation is essential before AISI is used in trauma pathways.

## Conclusions

In this single-center retrospective cohort, higher admission AISI was associated with ultrasound-detected in-hospital LEDVT before the earlier of discharge or post-injury day 30. AISI improved discrimination when added to clinical variables alone, whereas its incremental AUC gain was small and uncertain after coagulation markers were included. Internal validation showed calibration shrinkage, so AISI should be considered a complementary research marker, not a stand-alone clinical tool. Prospective external validation with standardized serial ultrasound, explicit post-discharge follow-up, and adjudicated incident VTE is required.

## Supporting information

S1 Data Analysis workbook containing primary models, candidate-parameter and events-per-parameter summaries, apparent and internally validated discrimination, Brier scores, calibration intercepts and slopes, sensitivity analyses, ultrasound summaries, supplementary covariate models, provenance fields, and locked quality-control outputs. (XLSX)

S2 Appendix Provenance-labeled screening, missingness, hospital-stay, follow-up, additional-covariate, and 23-record correction summaries. (DOCX)

S1 Checklist STROBE checklist for observational cohort studies. (DOCX)

S3 Data De-identified participant-level dataset with field definitions, participant follow-up, examination-level ultrasound records, prophylaxis timelines, screening-exclusion records, and quality-control checks. (XLSX)
