# Supplementary material for: Association of admission aggregate index of systemic inflammation with lower-extremity deep vein thrombosis after severe trauma
Source: PLoS One. 2026 Oct 21;21(10):e0360039. doi: 10.1371/journal.pone.0360039 (PMC13686453; doi:10.1371/journal.pone.0360039)
Supplement: Supplementary file 1 [file pone.0360039.s002.docx]

**S2 Appendix. Supplementary analyses and data-quality audit**

**Cohort assembly, missingness, follow-up, sensitivity analyses, and original-record quality control**

**Data sources and analytic scope**

All clinical values in this appendix derive from de-identified original medical records, imaging reports, treatment records, or screening logs. Quality-control re-abstraction updated specified chronology and ultrasound examination-count fields while retaining archived entries. No primary outcome or admission laboratory value was created, imputed, or altered.

**Provenance labels used in this appendix**

| **Data category** | **Definition** | **Permitted use** |
| --- | --- | --- |
| Recorded | Clinical field retained from the de-identified analysis dataset. | Primary and recorded-data analyses. |
| Re-abstracted | Specified field abstracted again from the original record; archived entry retained in the audit history. | Quality-control summaries and sensitivity analyses. |
| Calculated from original records | Variable calculated from directly recorded or re-abstracted clinical fields. | Supplementary descriptive and sensitivity analyses. |
| Combined recorded/re-abstracted | Analysis combining recorded outcomes and covariates with fields checked against original records. | Supplementary analyses; not a replacement for the prespecified primary model. |

**Appendix Table A1. Detailed disaggregation of the 74 screening exclusions**

| **Aggregate group** | **Specific exclusion reason** | **n** | **Data source** |
| --- | --- | --- | --- |
| Medication-related interference | Chronic immunosuppressive therapy | 6 | Original screening log |
| Medication-related interference | Pre-injury therapeutic anticoagulation | 10 | Original screening log |
| Special circumstance, prior DVT, or delayed transfer | Delayed transfer precluding baseline assessment | 8 | Original screening log |
| Special circumstance, prior DVT, or delayed transfer | Pregnancy | 3 | Original screening log |
| Special circumstance, prior DVT, or delayed transfer | Prior DVT | 5 | Original screening log |
| Systemic comorbidity affecting inflammation or thrombosis | Active malignancy | 8 | Original screening log |
| Systemic comorbidity affecting inflammation or thrombosis | Hematologic disorder | 5 | Original screening log |
| Systemic comorbidity affecting inflammation or thrombosis | Severe infection at admission | 7 | Original screening log |
| Unmet eligibility or insufficient outcome data | Age younger than 18 years | 3 | Original screening log |
| Unmet eligibility or insufficient outcome data | ISS lower than 16 | 6 | Original screening log |
| Unmet eligibility or insufficient outcome data | Missing admission CBC within 24 hours | 5 | Original screening log |
| Unmet eligibility or insufficient outcome data | Missing valid bilateral lower-extremity ultrasound outcome | 8 | Original screening log |

*The four aggregate exclusion groups (22, 16, 20, and 16) and all specific reasons are retained exactly as documented in the de-identified screening log.*

**Appendix Table A2. Limited included-versus-missing/excluded comparison**

| **Variable** | **Included (n=223)** | **Excluded subset with available fields (n=13)** | **Difference (95% CI)** | **SMD** | **Data source** |
| --- | --- | --- | --- | --- | --- |
| Age, years | 56.2 (16.5) | 54.1 (10.4) | -2.14 (-7.54 to 3.67) | 0.13 | Recorded |
| Male sex | 165/223 (74.0%) | 8/13 (61.5%) | -12.5 (-40.0 to 13.8) percentage points | 0.27 | Recorded |
| Injury Severity Score | 24.4 (6.6) | 22.8 (7.1) | -1.67 (-5.22 to 2.30) | 0.25 | Recorded |

*This limited comparison cannot identify the missingness mechanism or establish MCAR or MAR. No imputation was applied to the 223-participant analysis.*

**Appendix Table A3. Hospital length of stay, discharge, and follow-up summary**

| **Metric** | **Value** | **Denominator** | **Data source** |
| --- | --- | --- | --- |
| Total hospital length of stay, overall | 29.0 [24.0, 36.0] | 223 | Original medical records |
| Total hospital length of stay, no LEDVT | 27.5 [23.0, 33.8] | 130 | Original medical records |
| Total hospital length of stay, LEDVT | 32.0 [26.0, 39.0] | 93 | Original medical records |
| Hospitalized through day 30 | 109 | 223 | Original medical records |
| Discharged before day 30 | 114 | 223 | Original medical records |
| Post-discharge ultrasound | 0 | 223 | Original medical records |
| Documented post-discharge LEDVT detection | 0 | 223 | Original medical records |

*All documented ultrasound examinations and LEDVT events in the primary cohort occurred during hospitalization; no post-discharge event was counted.*

**Appendix Table A4. Primary and supplementary sensitivity models**

| **Analysis** | **n/events** | **AISI OR (95% CI)** | **P** | **AUC** | **Brier** | **Data source** | **Interpretation** |
| --- | --- | --- | --- | --- | --- | --- | --- |
| Primary Model 4 | 223/93 | 1.86 (1.26–2.73) | 0.002 | 0.829 | 0.158 | Recorded | Prespecified primary fully adjusted association model. |
| Add observed ventilation, surgery, pharmacologic prophylaxis | 223/93 | 1.91 (1.28–2.86) | 0.002 | 0.852 | 0.149 | Recorded | Exploratory post-baseline process adjustment; no causal interpretation. |
| Add original primary injury site | 223/93 | 1.81 (1.23–2.67) | 0.003 | 0.834 | 0.158 | Recorded | Observed injury-pattern sensitivity; primary injury site is not a fracture proxy. |
| Restrict to high-risk routine surveillance | 155/57 | 1.92 (1.17–3.16) | 0.010 | 0.855 | 0.137 | Recorded | Exploratory restriction to a more homogeneous recorded surveillance strategy. |
| Exclude 23 re-abstracted ultrasound-count records | 200/70 | 1.87 (1.17–2.97) | 0.008 | 0.851 | 0.139 | Recorded | Quality-control sensitivity; re-abstracted counts are used in the observation-process analysis. |
| Add pelvic/lower-extremity fracture | 223/93 | 1.84 (1.25–2.71) | 0.002 | 0.831 | 0.158 | Original medical records | Exploratory adjustment for fracture-related confounding. |
| Add fracture, bedbound status, and recorded ventilation | 223/93 | 1.94 (1.29–2.91) | 0.001 | 0.858 | 0.147 | Original medical records | Exploratory immobility and ventilation adjustment. |
| Add prophylaxis before outcome/discharge | 223/93 | 1.83 (1.25–2.69) | 0.002 | 0.830 | 0.158 | Original medical records | Time-ordered prophylaxis sensitivity; confounding by indication remains. |
| Add first ultrasound day and re-abstracted examination count | 223/93 | 1.81 (1.22–2.68) | 0.003 | 0.836 | 0.156 | Original medical records | Observation-process adjustment; informative surveillance may remain. |
| Fully adjusted supplementary model | 223/93 | 1.91 (1.26–2.89) | 0.002 | 0.864 | 0.144 | Original medical records | Exploratory supplementary model with increased overfitting risk. |
| First ultrasound day <=3 | 149/66 | 1.74 (1.12–2.70) | 0.014 | 0.827 | 0.164 | Recorded | Exploratory surveillance-timing stratum. |
| First ultrasound day >3 | 74/27 | 3.17 (1.24–8.08) | 0.016 | 0.871 | 0.129 | Recorded | Exploratory surveillance-timing stratum. |

*All supplementary models are exploratory and do not replace the prespecified primary Model 4. Odds ratios are per 1-SD increase in log-transformed AISI.*

**Appendix Table A5. Re-abstraction of 23 ultrasound examination counts**

| **Record ID** | **LEDVT** | **First US day** | **Last/positive day** | **Diagnosis day** | **Archived count** | **Re-abstracted count** |
| --- | --- | --- | --- | --- | --- | --- |
| EB010 | Yes | 2 | 5 | 5 | 1 | 2 |
| EB013 | Yes | 5 | 7 | 7 | 1 | 2 |
| EB027 | Yes | 3 | 7 | 7 | 1 | 2 |
| EB053 | Yes | 5 | 11 | 11 | 1 | 2 |
| EB077 | Yes | 2 | 5 | 5 | 1 | 2 |
| EB081 | Yes | 2 | 7 | 7 | 1 | 2 |
| EB093 | Yes | 6 | 7 | 7 | 1 | 2 |
| EB104 | Yes | 7 | 10 | 10 | 1 | 2 |
| EB105 | Yes | 3 | 5 | 5 | 1 | 2 |
| EB106 | Yes | 5 | 7 | 7 | 1 | 2 |
| EB117 | Yes | 3 | 7 | 7 | 1 | 2 |
| EB134 | Yes | 3 | 6 | 6 | 1 | 2 |
| EB137 | Yes | 3 | 8 | 8 | 1 | 2 |
| EB139 | Yes | 2 | 5 | 5 | 1 | 2 |
| EB144 | Yes | 5 | 10 | 10 | 1 | 2 |
| EB145 | Yes | 2 | 7 | 7 | 1 | 2 |
| EB188 | Yes | 7 | 13 | 13 | 1 | 2 |
| EB193 | Yes | 5 | 7 | 7 | 1 | 2 |
| EB200 | Yes | 2 | 4 | 4 | 1 | 2 |
| EB202 | Yes | 3 | 6 | 6 | 1 | 2 |
| EB207 | Yes | 3 | 4 | 4 | 1 | 2 |
| EB220 | Yes | 3 | 9 | 9 | 1 | 2 |
| EB224 | Yes | 2 | 7 | 7 | 1 | 2 |

*For all 23 records, the examination count was re-abstracted from the original ultrasound reports; archived counts remain in the audit history. The sensitivity analysis excluded these 23 records.*

**Appendix Table A6. Re-abstraction of five discharge and ultrasound chronology records**

| **Record ID** | **LEDVT** | **Endpoint day** | **Archived discharge day** | **Re-abstracted discharge day** | **Apparent post-discharge ultrasound (archived)** | **Post-discharge ultrasound after QA** | **Clinical conclusion** |
| --- | --- | --- | --- | --- | --- | --- | --- |
| EB009 | Yes | 28 | 26 | 28 | Yes | No | Discharge day was re-abstracted from the original record; the documented outcome remained inpatient. |
| EB020 | No | 22 | 21 | 22 | Yes | No | Discharge day was re-abstracted from the original record; the documented outcome remained inpatient. |
| EB060 | Yes | 20 | 19 | 20 | Yes | No | Discharge day was re-abstracted from the original record; the documented outcome remained inpatient. |
| EB135 | Yes | 24 | 22 | 24 | Yes | No | Discharge day was re-abstracted from the original record; the documented outcome remained inpatient. |
| EB165 | Yes | 18 | 16 | 18 | Yes | No | Discharge day was re-abstracted from the original record; the documented outcome remained inpatient. |

*Chronology corrections did not change LEDVT outcomes, admission laboratory values, or other clinical variables.*

**Appendix Table A7. Post-quality-control clinical-logic results**

| **Check** | **Observed** | **Requirement** | **Status** |
| --- | --- | --- | --- |
| source_patient_count | 223 | 223 | PASS |
| supplement_patient_count | 223 | 223 | PASS |
| source_verified_patient_count | 223 | 223 | PASS |
| first_us_after_discharge | 0 | 0 | PASS |
| last_positive_us_after_discharge | 0 | 0 | PASS |
| ledvt_diagnosis_after_discharge | 0 | 0 | PASS |
| event_after_discharge | 0 | 0 | PASS |
| positive_event_after_discharge | 0 | 0 | PASS |
| verified_count_source_report_mismatch | 0 | 0 | PASS |
| verified_count_archived_audit_mismatch | 0 | 0 | PASS |
| ledvt_event_outcome_mismatch | 0 | 0 | PASS |

*All prespecified count, chronology, outcome, and audit-reconciliation checks passed.*
