# Supplementary material for: Association of admission aggregate index of systemic inflammation with lower-extremity deep vein thrombosis after severe trauma
Source: PLoS One. 2026 Oct 21;21(10):e0360039. doi: 10.1371/journal.pone.0360039 (PMC13686453; doi:10.1371/journal.pone.0360039)
Supplement: Supplementary file 2 [file pone.0360039.s003.docx]

**S1 Checklist. STROBE statement—checklist of items that should be included in reports of cohort studies**

**Manuscript ID PONE-D-26-29357 | Completed 22-item checklist**

Association of admission aggregate index of systemic inflammation with lower-extremity deep vein thrombosis after severe trauma

| **Section / topic** | **Item** | **Official STROBE recommendation** | **Location in revised manuscript** | **Status** |
| --- | --- | --- | --- | --- |
| Title and abstract | 1a | Indicate the study’s design with a commonly used term in the title or the abstract. | Title; Abstract. | Reported |
| Title and abstract | 1b | Provide in the abstract an informative and balanced summary of what was done and what was found. | Abstract. | Reported |
| Introduction—Background/rationale | 2 | Explain the scientific background and rationale for the investigation being reported. | Introduction—paragraphs 1–4. | Reported |
| Introduction—Objectives | 3 | State specific objectives, including any prespecified hypotheses. | Introduction—final paragraph. | Reported |
| Methods—Study design | 4 | Present key elements of study design early in the paper. | Materials and methods—Study design and setting. | Reported |
| Methods—Setting | 5 | Describe the setting, locations, and relevant dates, including periods of recruitment, exposure, follow-up, and data collection. | Materials and methods—Study design and setting; Study population; Outcome definition and ultrasound assessment. | Reported |
| Methods—Participants | 6a | Give the eligibility criteria, and the sources and methods of selection of participants. Describe methods of follow-up. | Materials and methods—Study population; Outcome definition and ultrasound assessment; Fig 1. | Reported |
| Methods—Participants | 6b | For matched studies, give matching criteria and number of exposed and unexposed. | Not applicable; this cohort was not matched. | N/A |
| Methods—Variables | 7 | Clearly define all outcomes, exposures, predictors, potential confounders, and effect modifiers. Give diagnostic criteria, if applicable. | Materials and methods—Outcome definition and ultrasound assessment; Data collection and immune-inflammatory indices; Additional covariates and longitudinal data. | Reported |
| Methods—Data sources/measurement | 8* | For each variable of interest, give sources of data and details of methods of assessment (measurement). Describe comparability of assessment methods if there is more than one group. | Materials and methods—Data integrity and quality control; Outcome definition and ultrasound assessment; Data collection and immune-inflammatory indices. | Reported |
| Methods—Bias | 9 | Describe any efforts to address potential sources of bias. | Materials and methods—Data integrity and quality control; Statistical analysis; Discussion—limitations. | Reported |
| Methods—Study size | 10 | Explain how the study size was arrived at. | Materials and methods—Statistical analysis (fixed consecutive cohort and events-per-parameter statement). | Reported |
| Methods—Quantitative variables | 11 | Explain how quantitative variables were handled in the analyses. If applicable, describe which groupings were chosen and why. | Materials and methods—Data collection and immune-inflammatory indices; Statistical analysis. | Reported |
| Methods—Statistical methods | 12a | Describe all statistical methods, including those used to control for confounding. | Materials and methods—Statistical analysis. | Reported |
| Methods—Statistical methods | 12b | Describe any methods used to examine subgroups and interactions. | Materials and methods—Statistical analysis; Results—sensitivity analyses; Figs 4–5. | Reported |
| Methods—Statistical methods | 12c | Explain how missing data were addressed. | Materials and methods—Missing data and software; S1 Data—Missingness and QC sheets. | Reported |
| Methods—Statistical methods | 12d | If applicable, explain how loss to follow-up was addressed. | Materials and methods—Outcome definition and ultrasound assessment; Discussion—limitations. | Reported |
| Methods—Statistical methods | 12e | Describe any sensitivity analyses. | Materials and methods—Statistical analysis; Results—sensitivity analyses; Table 2; Fig 4. | Reported |
| Results—Participants | 13a* | Report numbers of individuals at each stage of study—e.g., numbers potentially eligible, examined for eligibility, confirmed eligible, included in the study, completing follow-up, and analysed. | Results—cohort assembly; Fig 1; S2 Appendix—Table A1. | Reported |
| Results—Participants | 13b* | Give reasons for non-participation at each stage. | Results—cohort assembly; Fig 1; S2 Appendix—Table A1. | Reported |
| Results—Participants | 13c* | Consider use of a flow diagram. | Fig 1. | Reported |
| Results—Descriptive data | 14a* | Give characteristics of study participants (e.g., demographic, clinical, social) and information on exposures and potential confounders. | Results—baseline characteristics; Table 1; S1 Data. | Reported |
| Results—Descriptive data | 14b* | Indicate number of participants with missing data for each variable of interest. | Materials and methods—Missing data and software; S1 Data—Missingness and QC sheets. | Reported |
| Results—Descriptive data | 14c* | Summarise follow-up time (e.g., average and total amount). | Materials and methods—Additional covariates and longitudinal data; Discussion—limitations; S2 Appendix—Table A3. | Reported |
| Results—Outcome data | 15* | Report numbers of outcome events or summary measures over time. | Results—ultrasound timing and outcome data; Fig 2; S1 Data. | Reported |
| Results—Main results | 16a | Give unadjusted estimates and, if applicable, confounder-adjusted estimates and their precision (e.g., 95% confidence interval). Make clear which confounders were adjusted for and why they were included. | Results—primary association and model performance; Tables 2–3; S1 Data. | Reported |
| Results—Main results | 16b | Report category boundaries when continuous variables were categorized. | Materials and methods—Statistical analysis. Continuous biomarkers were log-transformed and standardized; no outcome-guided categories were used. | Reported / N/A |
| Results—Main results | 16c | If relevant, consider translating estimates of relative risk into absolute risk for a meaningful time period. | Not presented because surveillance was not standardized; the outcome window and limitation are explained in Results and Discussion. | Explained |
| Results—Other analyses | 17 | Report other analyses done—e.g., analyses of subgroups and interactions, and sensitivity analyses. | Results—sensitivity analyses; Table 2; Figs 4–5; S1 Data; S2 Appendix. | Reported |
| Discussion—Key results | 18 | Summarise key results with reference to study objectives. | Discussion—opening paragraphs. | Reported |
| Discussion—Limitations | 19 | Discuss limitations of the study, taking into account sources of potential bias or imprecision. Discuss both direction and magnitude of any potential bias. | Discussion—limitations. | Reported |
| Discussion—Interpretation | 20 | Give a cautious overall interpretation of results considering objectives, limitations, multiplicity of analyses, results from similar studies, and other relevant evidence. | Discussion; Conclusions. | Reported |
| Discussion—Generalisability | 21 | Discuss the generalisability (external validity) of the study results. | Discussion—limitations and prospective agenda; Conclusions. | Reported |
| Other information—Funding | 22 | Give the source of funding and the role of the funders for the present study and, if applicable, for the original study on which the present article is based. | Online submission system (Financial Disclosure), as required by PLOS ONE. | Reported |

** Information is reported separately for participants with and without LEDVT where applicable.*

Official checklist source: STROBE Initiative, cohort studies checklist, https://www.strobe-statement.org/checklists/ (accessed 17 August 2026).
